# Investigation of SARS-CoV-2 Detection Method Applicability and Virus Occurrence in Food and Food Packaging

**DOI:** 10.17113/ftb.61.02.23.8018

**Published:** 2023-06

**Authors:** Zdenko Mlinar, Deni Kostelac, Ivančica Kovaček, Ana Klobučar, Vanja Tešić, Vedran Prahin, Jadranka Frece

**Affiliations:** 1 Andrija Štampar Teaching Institute of Public Health, Mirogojska cesta 16, 10000 Zagreb, Croatia; 2 Faculty of Food Technology and Biotechnology, University of Zagreb, Pierottijeva ulica 6, 10000 Zagreb, Croatia; 3 Faculty of Medicine, University of Rijeka, Braće Branchetta 20, 51 000 Rijeka, Croatia

**Keywords:** SARS-CoV-2, food, RT-PCR, coronavirus, foodborne transmission, RNA extraction

## Abstract

**Research background:**

While it is clear that SARS CoV-2 coronavirus is the primary respiratory virus, there are no entirely clarified ways of transmission. Foodborne transmission has remained an unexplained path. Therefore, the goals of this paper are to examine and present an assessment of the most appropriate of the four selected kits for RNA extraction for the testing and detection of SARS-CoV-2 on food packaging surfaces, food surfaces, and in food. This will enable to indicate the possibility of infection through contact or direct food consumption.

**Experimental approach:**

Finding the best technique is vital as RNA extraction is one of the essential elements in detecting SARS-CoV-2. This was achieved through an experiment with four commercial kits following the original manufacturers’ protocols, and with a modification of the original protocols that included the use of ethanol and isopropanol. The selected kit was used for RNA extraction from the swabs of packaging surfaces, food surface, and ready-to-eat food samples. The coronavirus was then identified using real-time reverse transcription-polymerase chain reaction (RT-PCR) assays to determine whether the SARS-CoV-2 virus or viral particles are present in the food chain with the overall purpose of demonstrating the possibility that food can contribute as a vehicle for the transmission of the virus.

**Results and conclusions:**

The findings of this investigation made the most effective extraction kit and protocol stand out. The results of the applicability of the kit indicated a significant share of positive samples of viral SARS-CoV-2 virus particles on surfaces from the environment where infected persons with 'silent' COVID-19 infection, with mild symptoms or no symptoms, were present. However, according to the findings of the second part of the study, the virus was not detected on the examined samples of food packaging surfaces, food surfaces, and food.

**Novelty and scientific contribution:**

The presented results distinguished one of the most suitable protocols for isolating RNA from environmental surface samples. The main contribution of the study is in the presentation of the results, that is, the examination of samples that are primarily related to the food chain, food packaging, food surfaces, and ready-to-eat food. The results of this study could also be helpful for further determination of the potential of food as a vector for the transmission of coronaviruses.

## INTRODUCTION

On 11 March 2020, the World Health Organization (WHO) declared the SARS-CoV-2 outbreak a global pandemic (*1*). The novel SARS-CoV-2 coronavirus led to the emergence of coronavirus disease (COVID-19), which caused over 6 million mortal cases, making it one of the deadliest pandemics in history. The WHO stated that it can spread from the mouth and nose in small liquid particles (*2*). However, during the SARS epidemic, direct contact with surfaces and faecal transmission was also reported (*3*). WHO points out that the SARS-CoV-2 virus can remain very stable at 4 °C, just like the SARS-CoV and Middle East respiratory syndrome (MERS) coronaviruses, that it is expected to behave similarly to its predecessors and that it could remain infectious at -20 °C for up to 2 years (*4*). In addition, many extensive studies confirm and clearly show the stability and variability of the persistence of SARS-CoV-2 and other coronaviruses in the environment, depending on different surfaces and the influence of climatic conditions on their stability (*5*–*7*). A couple of preliminary studies focus specifically on the importance of isolating and monitoring the occurrence of viruses in food and the stability of viruses on the surface of food, specifically salmon, shrimp, frozen chicken wings and pork (*5*, *8*–*12*).

Although complex transmission modes have not been fully understood, there are indications that the contamination of the seafood market could be the source of the COVID-19 outbreak in Wuhan, PR China (*12*). It is important to highlight that there are no documents that report the transmission through foods or packaging materials, but the capability of the virus to remain infectious on those matrices warns caution (*12*). The stability of SARS-CoV-2 in a wide pH range (pH=3–10) allows its stability in most food products (*12*). The study of Huang *et al.* (*13*) indicates that the oral cavity is an important site for SARS-CoV-2 infection and points to saliva as a potential route of SARS-CoV-2 transmission. Available literature suggests that more studies are necessary to better understand transmission modes, especially the role of food that has the potential to act as a vehicle for the mentioned virus (*12*). Field investigation in food retailers concluded that if preventative measures together with sanitizing protocols are employed, the risk of exposure to SARS-CoV-2 is low (*14*).

Most of the research since the beginning of the pandemic was focused on the ’mainstream’ processing of clinical specimens to detect the virus that is the primary causative agent of the COVID-19 disease. Therefore, at the time of this experiment, there were not any standardized protocols or methodology for detecting SARS-CoV-2 in the environmental samples available. Corman *et al.* (*15*) pointed out that the testing of samples should ideally be carried out in two steps, *i.e.* proving the presence of at least two gene sequences, just like the recommendation of the WHO guide (*16*). DNA amplification and detection methods take advantage of the conservation of the nucleotide sequence of a viral genome, which enables viral identification with excellent specificity and high sensitivity (*17*).

Based on the aforementioned, this study aims to assess, through comparative tests, the most appropriate and efficient of the four selected kits for RNA extraction, as well as to provide the most efficient solution for the testing and detection of SARS-CoV-2 *via* swabs of packaging surfaces, food surfaces, and ready-to-eat food on a wide range of food products available on the Croatian market during the pandemic. Although the SARS-COV-2 virus was not isolated from the samples taken from the food chain, the results of the experimental part of the research, in which the applicability of the kit was tested, indicated a significant proportion of positive samples from the environment where infected persons with less pronounced symptoms or no symptoms were present. Considering that similar studies presented in this paper showed the possibility of infection through contact or direct consumption of food, it can be concluded that there is a potential for infection, but it is negligible.

## MATERIALS AND METHODS

To test the presence of severe acute respiratory syndrome coronavirus 2 (SARS-CoV-2) on the surface of food, food packaging and in ready-to-eat food, it was necessary to evaluate the performance of commercially available kits for the isolation of viral ribonucleic acid (RNA).

### Isolation kit efficiency assessment

In order to determine the best isolation kit for the target RNA, kits from four manufacturers were used, designated as kit 1, 2, 3, and 4 (Table 1). Kit manufacturers included in the test are Agilent Technologies (La Jolla, CA, USA), Bioron Diagnostics GmbH (Römerberg, Germany), EuroFins Technologies (Freiburg, Germany), and Qiagen (Venlo, Netherlands), manufacturers are listed alphabetically, kit numbers are random, and have no relation to the order of the kits in the displayed table.

**Table 1:** Overview of extraction kits compared in the study

Manufacturer	*t*(storage)/°C	Regulatory status	Made in
Agilent Technologies	room	RUO	La Jolla, CA, USA
Bioron Diagnostics GmbH	room/2-8	CE-IVD	Romerberg, Germany
EuroFins Technologies	room	RUO	Freiburg, Germany
Qiagen	room	-	Venio, Netherlands

CE-IVD=European conformity label for in-vitro diagnostics, RUO=research use only

RNA was isolated according to the manufacturer’s instructions for each selected kit. Verification of kit procedures comprises implementation of the mouse norovirus, because the commercial standard of SARS-CoV-2 virus was not available for controlled contamination (murine norovirus, median tissue culture infectious dose assay (TCID_50_/mL ≈10^8^ copies/mL).

The standard is integral to VIR *Seek* Murine Norovirus (MNV), a quantitative real-time RT-PCR kit for food and environmental samples (EuroFins GeneScan Technologies, Freiburg, Germany). To determine the efficiency of PCR, equal volumes of MNV standard (10 µL) were added to the lysis buffer with varying virus dilutions (*N*(MNV)=undiluted, 10^-1^, 10^-2^ and 10^-3^). The assessment is based on the relative measure of the number of cycles at which the target analyte (after the RT-PCR reaction) curve intersects the threshold line, that is, the quantification cycle (C_q_) values and other calculations shown in the statistical processing of the results.

### Protocol modification based on ethanol/isopropanol use

In addition to the original protocol, testing was done with an alternative protocol in which ethanol (Scharlab, S.L., Sentmenat, Barcelona, Spain) or isopropanol (J.T. Baker, Deventer, the Netherlands) is used in the nucleic acid precipitation step. Modification within kits 1, 2 and 4, after sample lysis, was made during the RNA binding step; precisely equal volume of ethanol in the sample tube was replaced with equal volume of isopropanol, after which the procedure was the same according to the manufacturer’s protocol. Conversely, modification within kit 3 was also made after sample lysis, during the RNA binding step, but this time by adding equal volume of ethanol in the sample tube instead of equal volume of isopropanol.

### Confirmation of applicability of the selected kit

The applicability of the kit and method was confirmed under the conditions that guaranteed the presence of the virus, firstly by participating in proficiency testing, after which a pilot test of surface samples (*N*=84) was conducted. Pilot test samples were taken in quarantine areas where people were asymptomatic or had weakly expressed symptoms of the COVID-19 disease. Such a condition of infected persons is the basic assumption for the contamination of items, for instance, food packaging in the retail chain, which was discussed in many studies (*8*, *18*-*21*).

### Selection and sampling

Different categories of food were selected and sampled, direct food surfaces and packaging surfaces of packaged food: food surface samples (*N*=60) from the retail chain (packaged, unpackaged, fresh (*t*=4 °C), frozen (*t*=-20 °C), imported and domestic origin) and samples of ready-to-eat (RTE) meals (*N*=40), precisely 18 cold and 22 hot ready-to-eat meals. Swab samples were taken in the refrigerator and freezer of a large shopping centre as shown in Table S1.

From all surfaces of food samples or surfaces of food packaging (except ready-to-eat meals), a swab sample was taken according to the instructions of the ISO 15216-2:2019 standard (*22*) and the WHO practical guide (*16*). Surface samples were taken using plastic swabs with a synthetic tip, pre-soaked in a sterile phosphate-buffered saline (PBS) solution. Wooden sticks and cotton wool should be avoided as they may result in false negatives. The recommended wiping area is 25-100 cm^2^, but whenever possible, an area of 100 cm^2^ was taken to increase the chance of virus detection.

After labelling the test tubes with swabs, the samples were immediately transported in refrigerators to the laboratory for analysis. Samples of ready-to-eat meals were taken from the hot-meal department in several retail chains. In addition, samples were taken of cold and hot ready meals in bulk, where we know from the experience that the possibility of microbiological contamination is greater. The sampling procedure was carried out according to the instructions of the Nordic Committee on Food Analysis (NMKL) (*23*), CEN ISO/TS 17728:2015 (*24*) and CAC/GL 50-2004 (*25*). Samples of swabs and food were transported and kept at (5±3) °C until testing.

### RNA extraction from samples

The collected samples were processed using the methods that proved to be most effective, and RNA was isolated from the collected samples. Eluates obtained by extraction were used for a real-time reverse transcription-polymerase chain reaction (real-time RT-PCR).

### Real-time reverse transcriptase-polymerase chain reaction

Real-time RT-PCR starts with the reverse transcription (RT) of viral RNA into cDNA, which is amplified until the amplicon appears. EuroFins Technologies detection kits were used for this study, validated, and designed for testing environmental samples and food surfaces, which can detect envelope protein (E-gene), RNA-dependent RNA polymerase (RdRp gene, inside the Orf1ab polyprotein gene), and nucleocapsid protein (N1/N2 gene). All tests were performed in duplicate.

In the initial screening step, the isolated RNA was tested for the E-gene present in the SARS- and MERS-related coronaviruses using the VIR*Seek* SARS-COV-2 screen kit using specific SARS-CoV-2 nucleotide sequence primers (EuroFins Technologies). The kit was developed as an initial screening test to be used with the VIR*Seek* SARS-CoV-2 Ident 2 and VIR*Seek* SARS-COV-2 Mplex kits to confirm a positive screening test. The confirmatory test is performed by detecting the RdRp gene with the VIR*Seek* SARS-COV-2 Ident 2 kit (EuroFins Technologies) or the N-gene sequence (N1 and N2) with the VIR*Seek* SARS-COV-2 Mplex kit (EuroFins Technologies). Assay quality control assurance is provided by an internal positive control (IPC) for each reaction and using a positive (PC) and negative control (NC). According to these instructions, all positive samples were confirmed for at least one more target gene in this study.

RdRp and N gene PCR kit primer combinations are highly specific for SARS-CoV-2 and do not cross-react with SARS-CoV, MERS-CoV, or seasonal human coronaviruses HKU1, OC43, NL63 or 229E. In addition, primers from these kits do not cross-react with cDNA from other common foodborne viruses, including norovirus genogroups I and II, hepatitis A and E, rotavirus, adenovirus, or astrovirus (*17*).

The PikoReal™ Real-Time PCR System (Thermo Fisher Scientific Oy, Vantaa, Finland) was used for testing. The total reaction mixture for real-time RT-PCR was prepared from 5 μL of BasicMix and 15 μL of OligoMix to which 5 μL of the sample were added. The thermal profile for reverse transcription is 10 min at 50 °C, followed by enzyme activation and reverse transcriptase inactivation for 3 min at 95 °C.

Thermal cycling was performed at 40 cycles, denaturation for 3 s at 95 °C, annealing, and extension for 30 s at 58 °C. The channel through which fluorescence was detected for the targeted E-gene is FAM^TM^ and for the internal control Cy5^TM^. The results are displayed and evaluated through PikoReal™ software (*26*).

### Statistical data interpretation

The efficiency of the extraction kit is determined by the slope (b) of the line of the linear regression, from which the required level of efficiency (E) >90 % and R*^2^*>0.95 are defined.

The calculation formula for the slope is as follows:


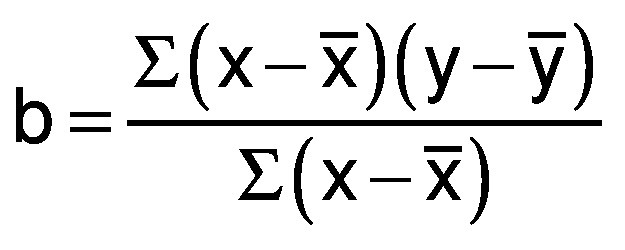
 /1/

The formula for the efficiency percentage calculation from the slope is:

E = -1 + 10^(-1/b)^ /2/

The formula for the coefficient of determination calculation:


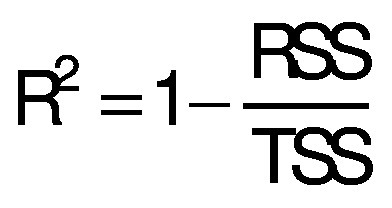
 /3/

where R^2^ is the coefficient of determination, RSS is the sum of squares of residuals and TSS is the total sum of squares.

The software GraphPad Prism (*27*) and Microsoft Excel (*28*) were used for calculations.

## RESULTS AND DISCUSSION

### Evaluation of performance characteristics of kits

The focus of the first part of the test was the extraction kits, *i.e.* the evaluation of the performance characteristics of four commercially available kits for isolating viral RNA. However, considering that these are environmental samples in which a large amount of targeted viral RNA was not expected, potential improvement of RNA isolation with the implementation of the formal, original protocol (O) set by the manufacturer was additionally tested with an alternative modified protocol (M). Untoro *et al.* (*29*) conducted a similar study where they compared methanol, chloroform, and 2-isopropanol as alternative solvents to ethanol in the isolation of dengue virus type 2 RNA, and concluded that the used methanol and 2-isopropanol gave better results than ethanol. Generally speaking, the processing of environmental samples leads to a specific problem. Lever *et al.* (*30*) emphasize that all environmental samples have their peculiarities and may require a specific fine adjustment of the used components and their ratios.

In a modification of the protocol within the kits where the original protocol is based on ethanol, isopropanol was used (kits 1, 2 and 4). Within the kit that uses isopropanol in the original instructions, alternatively, ethanol was used (kit 3). Namely, it has been shown that isopropanol is a more suitable solution in certain analyses, especially when a low concentration of RNA/DNA is expected. On the other hand, DNA is less soluble in solutions containing isopropanol than in solutions containing ethanol. Precipitation with isopropanol was performed at room temperature to reduce the risk of solutes such as sucrose or sodium chloride co-precipitating with DNA/RNA (*31*). The results in Table 2 show that three extraction kits gave satisfactory results, while they were absent in the case of kit number 4, and they were excluded from further evaluation. The alternative protocol gave significantly better results in the case of kit 2. Kit 3 gave the second-best result according to the original protocol, while the alternative protocol did not provide better results. Overall, kit 1 showed the best results when following the original protocol.

**Table 2:** Results of the C_q_ values of the tested extraction kits show differences in target extraction efficiency of MNV RNA whether ethanol (EtOH) or isopropanol (IPA) was used during the extraction

*N*(MNV)	C_q_							
Kit 1		Kit 2		Kit 3		Kit 4	
EtOH (O)	IPA (M)	EtOH (O)	IPA (M)	EtOH (M)	IPA (O)	EtOH (O)	IPA (M)
undiluted	26.30	29.99	33.04	30.29	31.72	28.94	37.83	38.57
10^-1^	29.02	33.53	35.81	32.92	33.96	31.48	40.44	41.50
10^-2^	32.49	37.03	39.96	36.59	37.49	34.51	NA	NA
10^-3^	36.60	40.35	43.29	39.96	40.79	39.33	NA	NA

MNV=murine norovirus standard solution (various dilutions), O=original protocol, M=modified protocol, C_q_=number of cycles at which the target analyte (after the RT-PCR reaction) curve intersects the threshold line

**Fig. 1:**
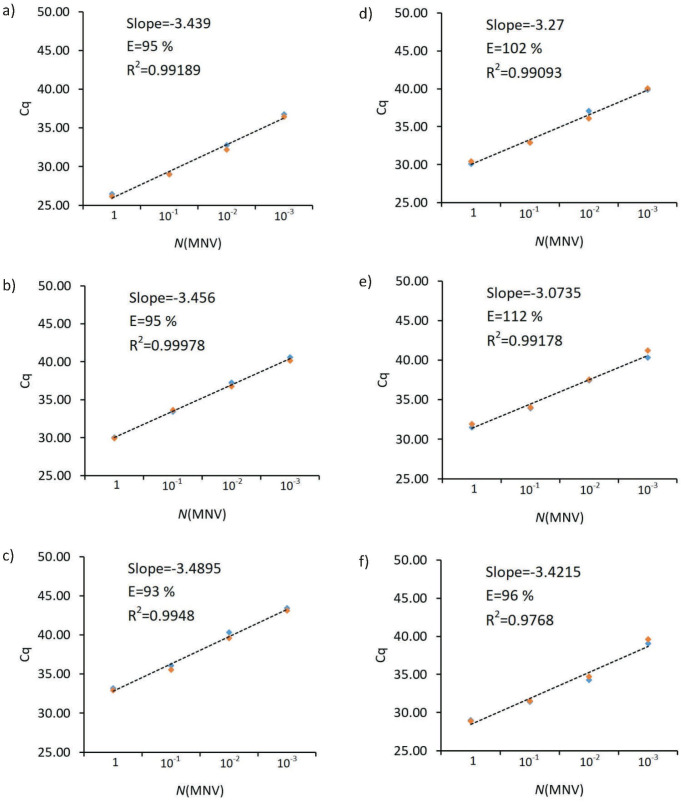
Presentation of the results of the efficiency test (E) of the three extraction kits tested by real-time RT-PCR detection of a double series of 10-fold dilution of the murine norovirus standard (MNV): a) kit 1 (ethanol), b) kit 1 (isopropanol), c) kit 2 (ethanol), d) kit 2 (isopropanol), e) kit 3 (ethanol) and f) kit 3 (isopropanol). C_q_=number of cycles at which the target analyte (after the RT-PCR reaction) curve intersects the threshold line

In addition, the efficiency (E) of the three extraction kits was tested by real-time RT-PCR detection of a duplicate series of a 10-fold dilution of the MNV standard. All tests showed satisfactory efficiency (E), while the R^2^ values of all kits except kit 3 (in which isopropanol was used) were >0.99, which meets the pre-defined required level (Fig. 1).

A similar study was conducted by O'Brien *et al.* (*32*), but for wastewater surveillance of SARS-CoV-2. These kits are specific in that they may contain additional inhibitor removal steps, so they are not directly comparable. In their study, Ambrosi *et al.* (*33*) also focused on extraction efficiency and dealt with protocol modification. In that study, proper evaporation of ethanol (to reduce downstream interference of the RT-PCR reaction) and extended incubation time during elution and centrifugation are highlighted as critical steps in RNA extraction. The mentioned modifications proved effective and are recommended for application in the study's kits to improve RNA recovery for automatic and manual extraction. Finally, a study by Ransom *et al.* (*34*) shows a comparison of three kits (systems) for automatic RNA extraction from clinical samples where it is clear that all three kits are satisfactorily efficient, but one stands out with slightly better results in terms of higher Cq values after the RT-PCR test. The authors conclude that this is likely the result of the use of half the elution volume and thus a higher RNA concentration. Also, there are similar studies by van Kasteren *et al.* (*35*) and Shen *et al.* (*36*), but about the evaluation of diagnostic kits. Very few studies have carried out this type of evaluation, and it is impossible to compare the results of this study directly.

### Confirmation of the suitability of the selected kits

The suitability of the selected extraction kit was double confirmed. The first confirmation of suitability was obtained by proficiency testing (LGC Standards Proficiency Testing, Bury, UK), during which three gene sequences: E, RdRp, and N, were successfully detected in the test sample (*37*). Furthermore, the method's suitability was confirmed through targeted pilot testing under conditions that guaranteed the presence of the virus (unpublished data). In particular, it was about testing surfaces in dedicated spaces (quarantine) where people who were asymptomatic or had weakly expressed symptoms of the COVID-19 disease were staying. At least two gene sequences were successfully confirmed in all samples.

**Fig. 2:**
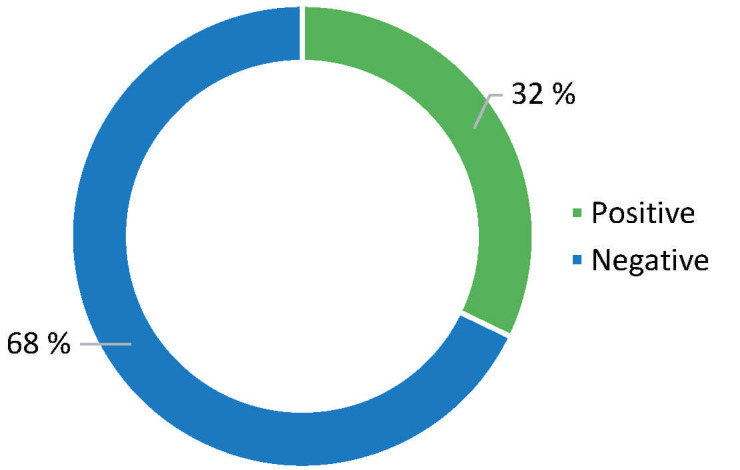
Ratio of total positive to negative samples from a targeted pilot testing of the presence of the SARS-CoV-2 virus or virus particles under conditions that guaranteed the presence of the virus

The overall results of the targeted pilot testing are shown in Fig. 2. Logical conclusion is that the amount of positive samples is significant (32 %) and those results point to how important the implementation of all prescribed hygiene measures as well as those concerning social distancing are.

Namely, the basic assumption and risk arise when such persons work in the preparation, serving, or in food traffic, as pointed out by the German Federal Institute for Risk Assessment (BfR) (*38*), stating that the coronavirus can be found on cutlery and dishes by direct sneezing or coughing of an infected person and cause infection if it reaches the mucous membrane of the nose or eyes. However, it also points out that they have not recorded such infections so far. This assertion is supported by the fact highlighted in several studies that viral RNA detected on positive food samples most likely originates from infected persons within the processing and distribution chain before packaging. The reason could be that food processing facilities are often identified as hotspots for COVID-19 due to overcrowded workplaces, close contact with colleagues, shared transportation or housing (*8*, *39*, *40*). For example, one study found that physical contact and sharing food during a conference in Singapore resulted in a group of people suffering from COVID-19 (*41*).

Ong *et al.* (*42*) monitored the air and areas where symptomatic patients with mild and moderate clinical symptoms were present and showed that as many as 87 % of the samples were positive. For example, in another study, the results of environmental samples in intensive care units (ICU) and COVID units were significantly more pronounced, 54 out of 57, *i.e.* 94.7 % in the ICU, and 9 out of 9, *i.e.* 100 % in the COVID unit (*43*).

### Examination of the presence of viruses on packaging surfaces, food surfaces and ready-to-eat food

After the applicability and suitability of the kit were confirmed by successful proficiency testing and targeted pilot testing under the conditions that guaranteed the presence of the virus, the kit was used in the testing of samples of food swabs and food packaging, which are considered to represent specific categories of food, taking into account diversity of the storage, storage temperature, and origin. As intended, RNA was extracted from all swabs and food samples (prepared meals), and the presence of the E-gene was detected using the VIR*Seek* SARS-CoV-2 Screen commercial kit. The results of all tested samples are summarized in Table 3.

**Table 3:** Presentation of the obtained results of testing surface swabs and ready-to-eat food

Target gene	Swab		Ready-to-eat meal	
Positive	Negative	Positive	Negative
E	0 (3 weeks positive)	60	0	40
RdRp	0 (1 week positive)	1	NA	NA
N	0	1	NA	NA

E=envelope protein, RdRp=RNA-dependent RNA polymerase, N=nucleocapsid protein, NA=not available

As for the screening kit, the settings of the automatic threshold limit and the criteria from the manufacturer's instructions were used (C_q_≤38). The test results of the swabs of the samples showed one positive and two more samples with C_q_>38. It should be noted that such high values are expected and usual in environmental samples. For example, in the study of Ong *et al.* (*42*), the average C_q_ value was 36.08. The samples that showed positive C_q_ values include all three samples from the category of fresh, refrigerated foods, *i.e.* fresh fruits and vegetables. All "positive" results have in common the absence of a characteristic sigmoid amplification curve, and as such, they cannot be considered fully valid results. Eventually, they can be characterized as weak positives. All "positive" samples, according to the recommendations, were additionally tested for the presence of the SARS-CoV-2 specific RdRP gene with the VIR*Seek* SARS-COV-2 Ident 2 kit. Only one sample had a positive result in terms of the obtained C_q_ value, but in this one too, the characteristic sigmoid amplification curve was absent. The same sample was also tested for the presence of the N gene with the VIR*Seek* SARS-COV-2 Mplex kit (EuroFins Technologies). No positive signal was recorded, *i.e.* no C_q_ value was expressed.

All 40 samples of the tested ready-to-eat meals were negative for E-gene presence. Generally speaking, the successful detection of viruses in food is a big challenge due to the physical and chemical properties of food, which include different matrices and the heterogeneous distribution of virus particles, low viral load, and very demanding isolation procedures (*11*, *44*), which is why care should be taken of potentially false-negative results or underestimated amounts of viruses. There are a large number of diseases caused by food and associated with viral infections, the most common of which are hepatitis A virus (HAV), hepatitis E virus (HEV), and norovirus (NoV), but other viruses, including enterovirus (EV), human rotavirus (RV), hepatitis E virus (HEV), astrovirus, Aichi virus, sapovirus, coronavirus, parvovirus, and human adenovirus have the potential for food transmission (*45*). In contrast, no evidence has been found that the ingestion of ready-to-eat food is the route of transmission of the SARS-CoV-2 virus, as evidenced by numerous studies and risk assessments (*8*, *19*, *44*, *46*-*50*). Most major food safety bodies, such as EFSA and FDA, have been obliged to clear any remaining doubts and closely monitor the situation. It is important to highlight that the early instances and outbreaks were linked to markets. Still, subsequent cases and outbreaks recurred in areas that were large warehouses of frozen food from many different parts of the world. The theory that the Huanan wholesale seafood market in Wuhan, PR China, was an early epicentre of the COVID-19 pandemic received its most considerable support yet in a comprehensive most recent study by Worobey *et al.* (*51*). However, it should not be forgotten that the sequencing of the genome of the SARS-CoV-2 virus isolated from the Xinfadi market in China confirmed that it is a European strain of the coronavirus (*52*), which turns the whole situation around a bit.

## CONCLUSIONS

From this study, it can be concluded that food and the food chain may have a more significant role in the SARS-CoV-2 pandemic than was initially thought. Numerous studies partially tried to confirm and explain the significance of a food and food transport role, particularly emphasizing the cold chain and generally the contact transmission. Among others, one of the main goals of this study was to define the most effective extraction kit and the procedure among the four selected commercially available kits. The study results showed that in some kits, using isopropanol instead of ethanol in the precipitation step can give a higher yield of RNA. The presented results made it possible to highlight the most efficient extraction kit for this type of study, and the applicability of the featured kit was further confirmed in two additional steps. In the first step, through proficiency testing, and in the second step through a pilot test for the detection of the SARS-CoV-2 virus on surfaces that guaranteed the existence of the virus or virus particles, *i.e.* in quarantine areas where asymptomatic persons or persons with mild symptoms of the disease were staying. The pilot test results showed that a significant proportion of the tested samples were positive for viral particles of the SARS-CoV-2 virus. Such results confirmed the justification of suspicion that similar results could be achieved if such asymptomatic persons or persons with less pronounced symptoms worked in the food chain. The second part of the study presented a relatively wide range of samples primarily related to the food chain, *i.e.* food packaging, food surfaces, and the ready-to-eat food. The final results of testing the presence of SARS-CoV-2 virus or viral particles on food packaging surfaces, food surfaces, and ready-to-eat food showed that the virus is not significantly present, and according to the obtained results, it could be concluded that food does not represent a significant risk and probability of infection with the SARS-CoV-2 virus. However, this possibility should not be ruled out considering the weak positive results.

Systematic monitoring of the virus is recommended to gain a better insight into the dynamics and possibilities of virus transmission through food, which is corroborated by the numerous studies mentioned in this paper. Two focus points need to be pointed out, firstly, the oral cavity is a significant site for SARS-CoV-2 infection, and its direct role in virus transmission through the oral cavity requires further research, and secondly, in legislative frameworks and risk reduction techniques, frozen and chilled foods are largely overlooked as possible vectors.

## Appendix

**Table S1:** Supplementary material list of analysed sample details

Sample no.	Sample name	Country of origin	Storage temperature	Packaging	Lot
1	White grapefruit	ZA	Chilled	Bulk	43-03
2	Williams pear (*l*=60 mm)	Serbia	Chilled	Bulk	L:31002238201029
3	Pomegranate variety Hicaz	Turkey	Chilled	Bulk	L:90450120TR
4	Cucumber variety Adrian	Spain	Chilled	Bulk	44/01
5	Tomato	Poland	Chilled	Bulk	43-06
6	Pepper mix variety CW	Spain	Chilled	Bulk	J26-THR
7	Zucchini variety Elite	Spain	Chilled	Bulk	44/04
8	Orange variety Valencia	ZA	Chilled	Bulk	44/04
9	Japanese apple persimmon	Spain	Chilled	Bulk	4501
10	Pineapple gold	Costa Rica	Chilled	Bulk	44/02
11	Apple variety Idared (*l*=70 mm)	Croatia	Chilled	Bulk	4305
12	Mandarin variety Satsuma	Croatia	Chilled	Bulk	0359
13	Apple variety Red delicious	Croatia	Chilled	Bulk	4501
14	Apple variety Jonagold	Croatia	Chilled	Bulk	L:99000099201028
15	Champignons (*m*=500 g)	Croatia	Chilled	Bulk	4406
16	Cabbage variety Coronet	Croatia	Chilled	Bulk	43-01
17	Paradise tomatoes	Croatia	Chilled	Packed	L031120
18	Chard variety Verca	Croatia	Chilled	Bulk	/
19	Crystal Bovary salad	Croatia	Chilled	Bulk	3010
20	Apple variety Gala	Croatia	Chilled	Bulk	4404
21	Breaded fish sticks	Croatia	Frozen	Packed	L040820 20:26
22	Quick-frozen chicken meat	Croatia	Frozen	Packed	10.10.2020./10.01.2021.
23	Breaded chicken dinosaurs	Croatia	Frozen	Packed	26.10.2020./26.10.2021.
24	Curry and beef roll	Croatia	Frozen	Packed	110420
25	Imperial vegetables (cauliflower, broccoli, carrots)	Croatia	Frozen	Packed	44124.42243
26	Chicken fries	Croatia	Frozen	Packed	27.10.2020/27.10.2021.
27	Pizza crispy pops	Croatia	Frozen	Packed	18.08.2020./18.08.2021.
28	Creperols (ham, cheese, cucumbers)	Croatia	Frozen	Packed	230620
29	Summer mix (peas, carrots, red pepper, potato, sweet corn)	Croatia	Frozen	Packed	43801,41407
30	Pie (chocolate/cherry)	Croatia	Frozen	Packed	/
31	Germknödel Toni Kaiser	Austria	Frozen	Packed	L 0284 11:52
32	Broiler duckling	Hungary	Frozen	Packed	L20124051
33	Mini natural baguette	Czech Republic	Frozen	Packed	L20125408 20125408
34	French salad	Belgium	Frozen	Packed	3:12 L20038-LN01
35	Patagonian squid	Morocco	Frozen	Packed	98/WOF/5219
36	Vegetable trio (cauliflower, broccoli, carrots)	Belgium	Frozen	Packed	08/04/22 18:57 L20099-LN01
37	Bulgur mixture	Spain	Frozen	Packed	16/10/2020 16 08:20
38	French fries	Belgium	Frozen	Packed	L G02 20 217 14:45
39	Breaded calamari rings	Spain	Frozen	Packed	30/09/2020 01:09
40	Seafood risotto	Belgium	Frozen	Packed	L1465350D
41	Long life milk *V*=1 L, *w*(mf)=2.8 %	Croatia	Chilled	Packed	23/01/21 23:27 000910K21
42	Yogurt *w*(mf)=2.8 %	Croatia	Chilled	Packed	14-20-102274
43	Chocolate hazelnut milk dessert	Croatia	Chilled	Packed	13.12.20. 10:10
44	Curd	Croatia	Chilled	Packed	2011 20L598
45	Solid yogurt *w*(mf)=3.2 %	Croatia	Chilled	Packed	80-20-102601
46	Long life milk *V*=1 L, *w*(mf)=3.5 %	Slovenia	Chilled	Packed	05.02.2021. 13:45:48 L6/U
47	Mozzarella	Czech Republic	Chilled	Packed	20 11 20 10:50 20 298 Kt
48	Fresh cream	Serbia	Chilled	Packed	28.11.2020. 01:00:50
49	Skyr	Slovenia	Chilled	Packed	29/11 00:27 29
50	Mayonnaise	Serbia	Chilled	Packed	05.2021 20:40 L02476839
51	Broccoli variety Parthenon	Spain	Chilled	Bulk	45/01
52	Paprika variety Vedrana	North Macedonia	Chilled	Bulk	44-03
53	Seedless watermelon	Brazil	Chilled	Bulk	44-02
54	Red California pepper	Spain	Chilled	Bulk	44/04
55	Apple variety Granny Smith	Croatia	Chilled	Bulk	L:990000992010
56	Pizza Margherita	Croatia	Frozen	Packed	L: 20247
57	Pizza picante	Croatia	Frozen	Packed	L: 20245
58	A cup of chestnut dessert premium	Croatia	Frozen	Packed	SN: 008294 Batch: 0003
59	Peanut butter	USA	Frozen	Packed	L00233OL60
60	Ice cream	France	Frozen	Packed	12/2021 6 05:18 L005E3 DOE02
